# Intestinal CCL25 expression is increased in colitis and correlates with inflammatory activity

**DOI:** 10.1016/j.jaut.2016.01.001

**Published:** 2016-04

**Authors:** Palak J. Trivedi, Tony Bruns, Stephen Ward, Martina Mai, Carsten Schmidt, Gideon M. Hirschfield, Chris J. Weston, David H. Adams

**Affiliations:** a National Institute for Health Research (NIHR) Birmingham Liver Biomedical Research Unit (BRU), Institute of Immunology and Immunotherapy, University of Birmingham, United Kingdom; b Liver Unit, Queen Elizabeth Hospital, Birmingham, United Kingdom; c Dept. of Internal Medicine IV, University Hospital Jena, Jena, Germany; d Center for Sepsis Control and Care, University Hospital Jena, Jena, Germany; e Department of General and Colorectal Surgery, Queen Elizabeth Hospital, Birmingham, United Kingdom

**Keywords:** Mucosal immunity, Lymphocyte recruitment, Inflammatory bowel disease, Primary sclerosing cholangitis, Immune-mediated liver disease

## Abstract

CCL25-mediated activation of CCR9 is critical for mucosal lymphocyte recruitment to the intestine. In immune-mediated liver injury complicating inflammatory bowel disease, intrahepatic activation of this pathway allows mucosal lymphocytes to be recruited to the liver, driving hepatobiliary destruction in primary sclerosing cholangitis (PSC). However, in mice and healthy humans CCL25 expression is restricted to the small bowel, whereas few data exist on activation of this pathway in the inflamed colon despite the vast majority of PSC patients having ulcerative colitis. Herein, we show that colonic CCL25 expression is not only upregulated in patients with active colitis, but strongly correlates with endoscopic Mayo score and mucosal TNFα expression. Moreover, approximately 90% (CD4^+^) and 30% (CD8^+^) of tissue-infiltrating T-cells in colitis were identified as CCR9^+^ effector lymphocytes, compared to <10% of T-cells being CCR9^+^ in normal colon. Sorted CCR9^+^ lymphocytes also demonstrated enhanced cellular adhesion to stimulated hepatic sinusoidal endothelium compared with their CCR9^–^ counterparts when under flow. Collectively, these results suggest that CCR9/CCL25 interactions are not only involved in colitis pathogenesis but also correlate with colonic inflammatory burden; further supporting the existence of overlapping mucosal lymphocyte recruitment pathways between the inflamed colon and liver.

## Highlights

• A significant proportion of tissue infiltrating T-cells express CCR9 in active colitis.
• Colonic CCL25 expression is upregulated in active colitis and correlates strongly with inflammatory burden.
• CCR9^+^ T-cells undergo adhesion to stimulated hepatic endothelium more readily than CCR9^−^ T-cells.

## 1 Introduction

Immune cell entry into tissues is a highly coordinated process controlled by the selective expression of cell adhesion receptors on circulating leucocyte subsets and their respective ligands within distinct vascular beds. In the gut, different intestinal sites show distinct patterns of constitutive chemokine expression, which compartmentalises different types of immune cells to particular regions. For example, under non-inflammatory conditions the chemokine CCL25 is expressed by small intestinal but not colonic epithelium, where it supports the recruitment of T-cells and B-cells expressing its receptor CCR9 [1]; whereas CCR10 and G Protein-Coupled Receptor (GPR)-15 are involved in recruiting IgA^+^ plasmablasts and T-cells, respectively, to the colon [2], [3], [4]. These findings suggest that expression levels of chemokines may shape regional differences in immune composition along the intestine. However most of these studies have been done in mice [5], [6], and there is a relative paucity of information about the roles of CCL25 and CCR9 in the human colon under inflammatory conditions. This is important as the CCR9/CCL25 pathway is being targeted to treat inflammatory bowel disease (IBD) and evidence of upregulation in active colitis would support clinical studies of anti-CCR9 therapy [7]. To this effect, a recently published randomised controlled trial in Crohn's disease demonstrated therapeutic benefit of on oral CCR9-antagonist over placebo only in the subset of patients with colonic involvement [8].

The intestinal and hepatic immune systems are intimately linked and venous drainage from the gut flows through the liver. The liver acts as a “firewall” and second site of immune regulation to deal with pathogens or antigens that escape immune control in the gut [9]. This close relationship probably explains why the liver is affected as an extra-intestinal site of injury in inflammatory bowel disease (IBD) [10]; exemplified by the fact that >80% of patients with the immune-mediated liver disease, primary sclerosing cholangitis (PSC), develop colitis [11]. One hypothesis to explain the clinical association between PSC and IBD suggests that mucosal effector T-cells are recruited to the liver in response to aberrant hepatic endothelial expression of adhesion molecules, which ordinarily are restricted to the gut [12], [13]. Although CCL25 is normally confined to the small intestine, we have reported strong expression on hepatic vessels in the inflamed liver, together with that of another gut-associated adhesion molecule, mucosal addressin cell-adhesion molecule (MAdCAM)-1 [12], [13]; which collectively drive recruitment of mucosal α4β7^+^CCR9^+^ effector T-cells from gut to liver in PSC [12], [13]. However, the IBD associated with PSC is typically a colitis [14], and previous studies have failed to detect tissue-infiltrating CCR9^+^ lymphocytes or mucosal CCL25 expression in the non-inflamed human colon [15], [16], [17], [18].

In an effort to ‘‘bridge the link” across the aberrant mucosal lymphocyte homing hypothesis [19], as well as our long-standing interest in exploring shared lymphocyte trafficking pathways that exist between gut and liver, the aims of this study were to determine activity of the CCR9/CCL25 axis in the human colon under inflammatory conditions.

## 2 Materials and methods

### 2.1 Intestinal tissue sampling

Intestinal tissue was obtained from patients undergoing surgery for refractory UC or stricturing colonic Crohn's disease. Control specimens were retrieved from ‘distal-to-tumour’ segments of non-IBD associated colorectal cancer (NC) – an absence of colonic inflammation being confirmed histologically. Colonic mucosal biopsies were obtained from patients with UC undergoing colonoscopic surveillance as part of routine standard of care. Human liver tissue was obtained through the Liver Unit at the Queen Elizabeth Hospital, from explanted livers removed at transplantation. Ethical approval was obtained from the local research and ethics committee and all patients granted full prior consent.

### 2.2 Preparation of tissue-infiltrating lymphocytes for flow cytometry

Mechanical digestion of tissue was carried out using the GentleMACS dissociator (Miltenyi Biotec Ltd, Surrey, UK) and mononuclear cell extraction performed via gradient centrifugation, as previously described [20]. The respective antibodies used to phenotype subsets and appropriate isotype-matched controls are provided in Supplementary Table 1. Samples were analysed in PBS/1 mM EDTA/2% FBS using a CyAn ADP 3-laser, 9-colour flow cytometer (Beckman Coulter Inc, Brea, CA, USA). CCR9 positive populations were defined by gating on live, CD3^+^, and either CD4^+^ or CD8^+^ cells. Dead cells were excluded using Near-IR live/dead-fixable dye (Life Technologies Ltd, Paisley, UK).

### 2.3 Fluorescence-activated cell (FAC)-sorting and flow-based adhesion assays

To obtain purified populations of T-cells for functional assays, antibody-labelled cells were sorted using a MoFlo XDP High-Speed Cell Sorter (Beckman Coulter Inc., Brea, California, USA) in purity mode.

Flow-based adhesion assays were conducted to compare the ability of sorted α4β7^+^CCR9^+^ versus α4β7^+^CCR9^–^ T-cells to adhere and transmigrate across stimulated human hepatic sinusoidal endothelial cells (HSEC) [21], [22], [23]. Briefly, HSEC were extracted from 150 g explanted liver tissue as previously described [21], [22], [23] and grown till confluence. At passage 3–4, HSEC were transferred to micro-capillaries for further culture, and when a confluent monolayer was evident they were stimulated with TNFα and methylamine as described by Liaskou et al. [22], prior to perfusion of purified T-cell populations at a shear stress of 0.05 Pa. T-cells that had adhered to HSEC were visualized by phase contrast microscopy (10 × objective) and classified as static (firmly adherent) or migrated cells. Total adhesion was calculated as cells/mm^2^ normalised to the number of lymphocytes perfused [23].

#### 2.3.1 Protein immunoprecipitation and western blotting

Protein was extracted from 60 to 100 mg snap-frozen tissue by incubation in ice-cold lysis buffer (CellLytic MT – Sigma–Aldrich Ltd, Dorset, UK) containing protease inhibitor (cOmplete Mini – Roche, Indianapolis, USA), phosphatase inhibitor (phosSTOP – Roche) and DNase I (Roche). Lysate protein concentrations were determined against a protein standard using a bicinchoninic acid (BCA) assay and normalised to 2 mg ml^−1^.

Given that chemokines are often found at low levels in tissues with a propensity to self-aggregate and multimerise [24], an immunoprecipitation step (IP) was incorporated to enrich and purify protein lysates. In brief, 0.6 mg of protein G Dynabeads^®^ (Life Technologies Ltd., Paisley, UK) were incubated with capture antibody (goat polyclonal anti-human CCL25; Cat No.: AF334 – R&D systems, Minneapolis, USA) at ambient temperature under continuous rotation (1 h) and the sample lysate added (continuous rotation; 1 h). Magnetic separation was used to obtain bead bound protein, and non-bound cleared lysate kept for analysis of housekeeping protein and as a negative internal control for the primary target. The remaining bead-bound sample was washed and subjected to temperature dissociation (70 °C, 20 min) to remove beads prior to resuspension in SDS-PAGE sample buffer (200 mM Trizma Base *p*H 6.8, 20% glycerol, 10% SDS, 0.05% bromophenol blue, 10 mM β-mercaptoethanol).

Immunoprecipitated protein or cleared lysate (20 μg) from each sample was resolved on a 12% SDS–PAGE gel and transferred to a nitrocellulose membrane. Membranes were blocked in 5% non-fat milk dissolved in PBS-Tween20 at ambient temperature (1 h) and subsequently incubated overnight at 4°C with primary detection antibody (mouse raised against human CCL25: Clone 52529 – R&D systems; or clone 1.2_4G1-1G4-1C9 – Peprotech, New Jersey, USA) or housekeeping protein (mouse raised against human β-actin: clone AC-74 – Sigma) diluted in 5% milk/PBS-Tween20. The following day, membranes were incubated with horseradish peroxidase conjugated anti-mouse secondary antibodies (1 h; ambient temperature) (Dako Ltd., Cambridge, UK; 1/2500 dilution). Protein bands were detected with the PicoWest ECL system (Thermo Fisher Scientific Inc., Rockford, USA). Membranes were not stripped.

#### 2.3.2 Enzyme-linked immunosorbent assay (ELISA)

ELISA for detection of CCL25 was performed on protein lysates from resected tissue according to manufacturers instructions (2bScientific Ltd., Upper Heyford, UK).

### 2.4 Gene-expression studies

RNA was extracted from snap-frozen tissue samples using the Qiagen RNeasy mini-kit with on column DNase digestion (Qiagen GmBH, Hilden, Germany). Quantification and purity of RNA was determined by UV absorbance at 260 nm and 280 nm (Implen Nanophotometer, Geneflow Ltd., Lichfield, UK) and concentration adjusted to 50 μg ml^−1^. One microgram of RNA was reverse-transcribed using the iScript cDNA synthesis kit (Bio-Rad Inc, Hercules, USA). Differences in target mRNA expression between UC and NC was determined in triplicate for each sample by quantitative real-time PCR (qRT-PCR) (Roche Lightcycler 480; TaqMan Assay Mix, Life Technologies). All primers and probes spanned exon–exon junctions and were pre-designed and obtained from Life Technologies (Supplementary Table 2). For relative quantification analysis, data were normalised to the appropriate housekeeping gene using ‘E-analysis’ (Roche) and the device software (version 1.5.0.39). We have recently determined GUS-β as being highly conserved by GeNORM and Bestkeeper algorithms in normal colon as well as colonic cancer [20]. Indeed, GUS-β illustrated stable expression between UC and NC specimens, as well as across ordinal degrees of colonic inflammatory activity (data not shown), and was therefore considered a suitable housekeeping gene for assessment in this context.

### 2.5 Statistical analysis

The distribution of continuous variables was tested using the Kolmogorov–Smirnov test. Non-parametric data are presented as median and interquartile range (IQR); or mean and standard deviation (SD) if confirming to a normal distribution. The Mann–Whitney (MW) test and the Kruskal-Wallis test were conducted when comparing between two or more independent groups, respectively. Non-parametric measures of statistical dependence between two continuous variables were conducted using Spearman's rank correlation coefficient.

## 3 Results

### 3.1 CCR9^+^ T-cells are present in inflamed but not normal human colon

**Fig. 1:**
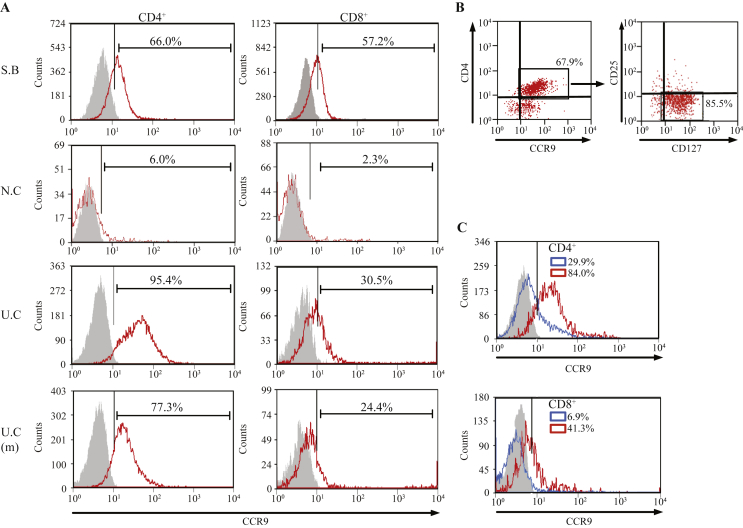
CCR9 Expression of Tissue-Infiltrating T-Cells in Small and Large Bowel. Representative flow cytometry plots illustrating [A] percentage CCR9 expression on intestinal CD4^+^ and CD8^+^ T-cells in terminal ileum/small bowel (SB; *n* = 5), non-inflamed colon (NC; *n* = *5*), ulcerative colitis (UC; *n* = 6) and UC resection specimens without evidence of macroscopic inflammation (UC (m); *n* = 4). Fluorescence minus one (FMO) controls were used to determine specificity of staining for CCR9: red histograms indicate positive antibody staining and grey, the isotype-matched control. CD4^+^ CCR9^+^ T-cells infiltrating the inflamed colon expressed high levels of CD127 but little CD25 [B]. The proportions of CD4^+^ and CD8^+^ CCR9^+^ T-cells were also determined in Crohn's resection specimens [C] (*n* = 2); wherein, red histograms indicate active inflammatory stricturing, blue histograms represent adjacent areas of non-active disease, and grey the isotype-matched controls (from areas of active stricturing). All plots are gated on live, CD3^+^ cells.

Consistent with prior reports, we found that 62.4% (SD ±19.7%) of tissue-infiltrating CD4^+^ and 68.2% (±12.4%) of CD8^+^ T-cells were CCR9 positive in the terminal ileum/small bowel (Fig. 1A**)** whereas very few CCR9^+^ T-cells were detected in normal colon (5.5 ± 0.5% and 4.9 ± 2.4%, respectively). In contrast, specimens from patients with ulcerative colitis refractory to medical therapy contained significantly more CCR9 expressing CD4^+^ (92.6 ± 4.1%; *p* < 0.001) and CD8^+^ T-cells (34.3 ± 3.8%; *p* = 0.013) than non-inflamed colon. Increased frequencies albeit at lower levels were also seen in large bowel specimens with only microscopic colitis. The majority of CD4^+^ CCR9^+^ T-cells were CD127^+^, with little or no expression of CD25. Collectively, these findings suggest that effector CCR9^+^ lymphocytes, but not regulatory T-cells (T_reg_), infiltrate the inflamed colon (Fig. 1B).

Phenotypically, UC is characterised by continuous involvement of the colon starting at the rectum and showing variable proximal extension [25]. Crohn's disease on the other hand is typified by the presence of skip lesions in which regions of active disease are interspersed with segments free of inflammatory involvement [26]. To test the hypothesis that colonic CCR9^+^ lymphocytes are restricted to areas of active inflammation, we compared tissue-infiltrating T-cell populations in resected Crohn's inflammatory strictures with those in neighbouring segments of uninvolved colon (Fig. 1C). In the cases studied, >80% of CD4^+^ and >40% of CD8^+^ T-cells in regions of active inflammatory stricturing were CCR9 positive compared with ∼30% and ∼7% in non-inflamed colon from the same patients.

### 3.2 α4β7^+^CCR9^+^ T-cells undergo adhesion and transmigration across HSEC

We have previously shown that HSEC can support the recruitment of gut-tropic α4β7^+^ lymphocytes, following endothelial stimulation with TNFα and methylamine [22]. Extending these observations further, a significantly greater proportion of FAC-sorted α4β7^+^CCR9^+^ T-cells underwent adhesion to stimulated HSEC under flow, relative to that observed for α4β7^+^CCR9^–^ T-cells (Supplementary Fig. 1).

### 3.3 Colonic CCL25 expression is upregulated in colitis

**Fig. 2:**
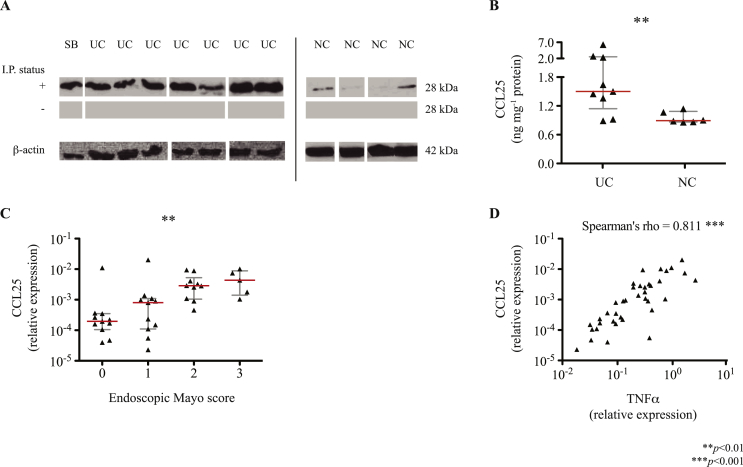
Colonic CCL25 Expression under Non- and Pro-Inflammatory Conditions. CCL25 protein expression was determined by western blots post-immunoprecipitation, I.P. [A], probing for CCL25 in tissue lysates of resected terminal ileum/small bowel (SB), large bowel afflicted with ulcerative colitis (UC) and non-inflamed colon (NC). Band sizes in keeping with dimeric CCL25 are illustrated for samples probed following I.P. (+), the paired cleared lysate (−), and β-actin (housekeeping; cleared lysates only). Membranes bearing NC samples were subject to greater exposure times given the relatively low abundance/absence of CCL25 under non-inflammatory conditions and are therefore shown as a separate group. Differential expression of CCL25 in tissue samples was confirmed by ELISA [B]. Correlation with inflammatory burden was assessed in mucosal biopsy samples obtained during routine, surveillance colonoscopy (*n* = 37) by qRT-PCR. Given that CCL25 gene expression was not detectable in non-inflamed colon (control), data are presented relative to GUS-β (housekeeping gene) across endoscopic Mayo severity scores [C] and in correlation with TNFα expression [D]. Asterisks are indicative of statistically significant differences.

Having demonstrated a population of tissue-infiltrating CCR9^+^ T-cells in the inflamed colon, we proceeded to look for its cognate ligand. Indeed, CCL25 mRNA expression was detected in resected colonic tissue from patients with refractory colitis (*n* = 10; median expression relative to GUS-β: 9.6*ε* ^−4^; IQR 2.0*ε* ^−4^ – 3.2*ε* ^−3^) but not in normal colonic tissue (*n* = 10). These differences were reflected in increased CCL25 protein expression measured by immunoprecipitation and western blotting (Fig. 2A), and confirmed using ELISA analysis of the same tissue protein lysates (Fig. 2B).

### 3.4 CCL25 expression in the colon correlates with severity of colitis

To determine whether CCL25 expression is detectable in colitis during earlier stages of inflammation, mucosal biopsies were obtained from individuals with ulcerative colitis undergoing colonoscopy as part of routine standard of care (kindly provided by University Hospital Jena (Germany); Table 1). Although mucosal biopsies are not large enough for protein or single cell flow-cytometry analysis, we were able to quantify CCL25 mRNA expression and demonstrate a significant correlation with both mucosal inflammation as assessed by the endoscopic Mayo score, and colonic TNFα transcription as an alternative marker of inflammatory activity (Fig. 2C and D).

No significant differences in CCL25 expression were revealed between colonic biopsy site, treatment status with aminosalicylates, immunosuppressive exposure (stratified according to use of steroids, thiopurines, calcineurin inhibitors, biological therapy) or patient age (data not shown).

## 4 Discussion

Evidence form murine studies suggest that CCL25 and CCR9 are involved in lymphocyte recruitment to the small intestine but not the colon. We now show that although CCL25 is largely absent from non-inflamed human colon, expression is markedly upregulated in colitis and correlates with inflammatory activity. Moreover, CCL25 expression in the colon is associated with high frequencies of CCR9^+^ tissue-infiltrating effector T-cells in patients with colitis, which exhibit increased potential toward adhesion to liver endothelium. These findings are important for several reasons: firstly they support a role for CCL25 expression and CCR9^+^ effector cells in colitis and show that CCR9-dependent recruitment is not confined to regulatory cells; secondly they suggest that CCR9 is a potential therapeutic target in UC as well as colonic Crohn's disease [8], [27], and thirdly they support a pathogenic role for effector T-cells activated in the colon, which have also been identified as being pro-inflammatory in PSC liver [12].

CCR9 is expressed on nearly all intraepithelial lymphocytes (IELs) and lamina propria lymphocytes (LPLs) in the jejunum, and at high frequencies in the terminal ileum [15]. In contrast, relatively few T-cells are reportedly CCR9 positive in the colon or mesenteric lymph nodes (MLN) [18]. Moreover, Papadakis *et al*. found low frequencies of CD4^+^CCR9^+^ T-cells in the peripheral blood of patients with colonic as opposed to small bowel Crohn's disease [28]. The same group reported low frequencies of CD3^+^CCR9^+^ cells in normal human colonic mucosa, consistent with our findings [18]. However, none of these studies looked in detail at inflamed human colon. We not only detected elevated frequencies of CCR9^+^ T-cells in active macroscopic colitis but also saw high (albeit relatively fewer) numbers in microscopic colitis. Taken together, our findings imply that activation of this pathway takes place early in colitis and is upregulated with disease activity [29].

*Ccl25* gene expression is restricted to the small intestine in uninjured wild type mice [16], [30], [31] as well as the Samp1/YitFc model of IBD [32]. Similar results are reported in the *TnfΔ*^*ARE*^ model of small bowel Crohn's disease [33], *Rag2*^−/−^ mice [34] and studies in higher order mammals [35]. Nevertheless, certain studies do report colonic *Ccl25* transcripts in spontaneous murine models of colitis [36], as well as colonic inflammation induced by dextran sulphate sodium [37], [38] and oxazolone [39]. We extend upon these observations by showing a striking positive correlation between human colonic *CCL25* gene expression and inflammatory indices across two patient cohorts with ulcerative colitis, in association with detectable CCL25 protein levels and a CCR9^+^ colon-infiltrating effector T-cell population. These findings may also contribute to the colonic cancer risk in IBD which relates to inflammatory burden, given the ability of CCR9/CCL25 interactions to mediate colonic tumour growth, invasion and metastasis [40].

The potential to imprint gut-tropism onto lymphocytes was long-believed as restricted to intestinal dendritic cells (DC) within the lamina propria and MLN [41], although works from the Blizzard institute (London, UK) have recently identified DC and CD14^+^ macrophages from the inflamed human colon as also possessing such capabilities [42]. Moreover, murine studies suggest that HSEC under certain circumstances can imprint gut-tropism through the generation of α4β7^+^CCR9^–^ T-cells. However, under such circumstances ‘HSEC-primed’ T-cells are dominated by regulatory functions, rather than ‘gut-primed’ α4β7^+^CCR9^+^ T-cells which exhibit an effector phenotype [43], [44], [45]. Our data builds upon these findings by showing how α4β7^+^CCR9^+^ T-cells undergo enhanced adhesion and transmigration across stimulated liver endothelium compared with their α4β7^+^CCR9^–^ counterparts. In view of the evolving use of anti-CCR9 therapies in IBD, these findings support therapeutic exploration in models exhibiting concomitant features of cholangitis and colitis [45], [46]; of particular relevance given that it is the CCR9 positive subset of mucosal T-cells which are implicated in the pathogenesis of PSC [12].

If CCL25 expression is driven by colitis activity, and colonic CCR9^+^ T-cells are responsible for driving hepatobiliary inflammation in PSC, then it should follow that the risk of PSC increases with intestinal activity; an observation which does not hold true clinically. However, pre-exposure of primed CCR9^+^ T-cells to high CCL25 levels in the gut during the onset of active IBD, may be capable of modulating subsequent migratory responses [47]. In such a model, pro-inflammatory, effector mucosal CCR9^+^ T-cells would be preferentially recruited to the gut during the onset of active colonic disease in response to high levels of intestinal CCL25 expression. Thereafter, mucosal T-cells would down-regulate expression of active CCR9 (“chemokine desensitisation”), favouring local retention in the gut for as long as colonic inflammation persists. On achieving remission from a colitis flare, the colonic CCL25 gradient is attenuated, and primed mucosal CCR9^+^ lymphocytes now become permissive to recruitment to the liver sinusoids in response to aberrant hepatic endothelial CCL25 expression – as observed in the PSC liver [12]. Of interest, the risk of PSC disease recurrence following liver transplantation appears greatest in patients with IBD who retain an intact colon [48], [49], [50]. However, the precise factors regulating endothelial expression of CCL25 in the native (and indeed transplanted) liver remain elusive, and command further investigation.

In summary, we report the involvement of CCL25 and CCR9 effector T-cells in colonic inflammation, providing further evidence to support a role for CCR9 in lymphocyte homing to the large bowel. Given the strong links between hepatobiliary inflammation and the presence of IBD, these findings when taken together with our previously published data, support a role of CCR9/CCL25 interactions in driving recruitment of mucosal effector cells to the gut as well as liver in patients with ulcerative colitis.

## Grant support and funding

PJT, CW, SW, GMH and DHA all received funding from the NIHR BRU PJT is funded by a Wellcome Trust Clinical Fellowship Award (099907/Z/12/Z) TB and MM received funding from the Federal Ministry of Education and Research (BMBF) Germany (FKZ: 01 E0 1002). TB receives funding from the German Research Foundation (DFG) Germany (FKZ: BR4182/3-1).

## Disclosures

This article presents independent research funded by the NIHR. The views expressed are those of the authors and not necessarily those of the NHS, the NIHR or the Department of Health.

## Competing interests

None.

## Figures and Tables

**Figure:**
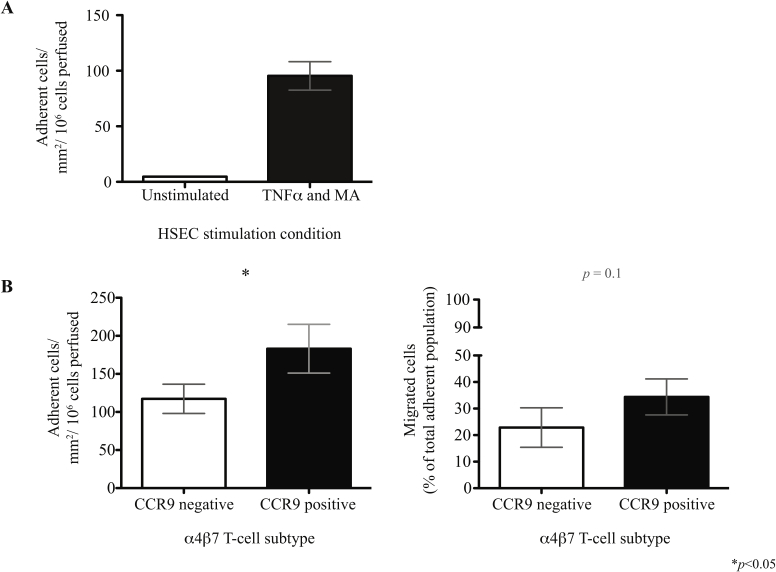


**Table 1:** Characteristics of patients undergoing colonoscopic surveillance (n = 37).

Patient characteristic	Number of patients (%)
Median age (IQR)	38yrs. (26–46)
Male; N (%)	22 (59)
**Biopsy site; N (%)**	
- rectum	13 (35)
- sigmoid	16 (43)
- descending colon	3 (8)
- transverse colon	1 (3)
- ascending colon	2 (5)
- caecum	2 (5)
**Endoscopic Mayo score; N (%)**	
0	11 (30)
1	11 (30)
2	10 (27)
3	5 (14)
**Primary sclerosing cholangitis; N (%)**	3 (8)
**Aminosalicylates**	
- Oral	29 (78)
- Topical	2 (5)
**Immunosuppression; N (%)**^a^	
- Any	21 (57)
- Corticosteroid (oral)^b^	9
- Corticosteroid (topical)^c^	3
- Anti-proliferative^d^	8
- Biologic^e^	10
- Calcineurin inhibitor	1

a Includes those on combination therapy with >1 agent.

b Prednisolone was utilised in all cases.

c Budesonide or hydrocortisone.

d Azathioprine, 6-Mercaptopurine of Methotrexate.

e Infliximab or Adalimumab.
