# Supplementary material for: Intestinal CCL25 expression is increased in colitis and correlates with inflammatory activity
Source: J Autoimmun. 2016 Apr;68:98–104. doi: 10.1016/j.jaut.2016.01.001 (PMC4803021; doi:10.1016/j.jaut.2016.01.001)
Supplement: Supplementary file 1 [file mmc1.docx]

| **Supplementary Table 1: Antibodies Used for Flow Cytometry** | | |
| --- | --- | --- |
| **Conjugated antibodies** | **Supplier** | **Isotype**  **(clone)** |
| Mouse anti-CD3 PE-Cy7 | BioLegend | IgG1  (SK7) |
| Mouse anti-CD4 V500 | BD Horizon | IgG1  (RPA-T4) |
| Mouse anti-CD4 FITC | BD Horizon | IgG1  (RPA-T4) |
| Mouse anti-CD8 PE-CF594 | BD Horizon | IgG1  (RPA-T8) |
| Mouse anti-CD127 PerCPCy 5.5 | BD Pharmingen | IgG1  (HIL-7R-M21) |
| Mouse anti-CD127 FITC | BD Pharmingen | IgG1  (HIL-7R-M21) |
| Mouse anti-CD127 APC | eBioscience | IgG1  (eBioRDR5) |
| Mouse anti-CD25 Pac. Blue | BD Pharmingen | IgG1  (M-A251) |
|  α4 integrin APC | BD Biosciences | IgG1  (9F10) |
| Rat anti-β7 integrin PE | BD Pharmingen | IgG2a  (FIB504) |
| Mouse anti-CCR9 AF 488 | BD Pharmingen | IgG2a  (112509) |
| Mouse anti-CCR9 AF 647 | BD Pharmingen | IgG2a  (112509) |
| **Isotype-matched control (IMC)** | |  |
| Mouse PE-Cy7 | BD Pharmingen | IgG1  (MOPC-21) |
| Mouse V500 | BD Horizon | IgG1  (X40) |
| Mouse FITC | BD Horizon | IgG1  (X40) |
| Mouse PE-CF594 | BD Horizon | IgG1  (X40) |
| Mouse PerCPCy 5.5 | BD Pharmingen | IgG1  (MOPC-21) |
| Mouse Pac. Blue | BD Pharmingen | IgG1  (MOPC-21) |
| Mouse APC | BD Pharmingen | IgG1  (MOPC-21) |
| Rat PE | BD Pharmingen | IgG2a  (R35-95) |
| Mouse AF 488 | BD Pharmingen | IgG2a  (G155-178) |
| Mouse AF 647 | BD Pharmingen | IgG2a  (G155-178) |

| **Supplementary Table 2: Primer/Probe Mixes for qRT-PCR** | |
| --- | --- |
| **Target** | **TaqMan Assay I.D.** |
| *CCL25* | Hs00608373_m1 |
| *TNFα* | Hs01113624_g1 |
| *GUS-β* | Hs00939627_m1 |

Cycling conditions were as follows: 95^o^C for 10 seconds, 59^o^C for 30 seconds and 72^o^C for 1 second.

**Supplementary Figure 1: Stimulated Hepatic Endothelial Cells Support Adhesion of CCR9^+^ T-cells under Flow**

CD3^+^α4β7^+^ cells were sorted from peripheral blood and then perfused over a monolayer of hepatic sinusoidal endothelial cells (HSEC) at flow rates observed in the human liver (shear stress (0.05 Pa). HSEC were either left unstimulated, or treated with TNFα (20 ) and methylamine (100 μM) for 4 hours as previously described [21]. The degree of adhesion experienced by gut-tropic α4β7^+^ T-cells as a whole is presented for unstimulated (control) versus stimulated HSEC in [A]. Differences in adhesion and percentage transmigration between α4β7^+^CCR9^+^ T-cells versus α4β7^+^CCR9^–^ T-cells across HSEC are shown as subtypes in [B] (stimulated endothelium only).

Plots shown are representative of the mean (+SD) of pooled data from at least 10 technical replicates per independent experiments (*n=*3). Asterisks are indicative of statistically significant differences between groups.
